# TEMPI Syndrome: Update on Clinical Features, Management, and Pathogenesis

**DOI:** 10.3389/fendo.2022.886961

**Published:** 2022-05-19

**Authors:** Jian Xu, Wenqi Liu, Fengjuan Fan, Bo Zhang, Fei Zhao, Yu Hu, Chunyan Sun

**Affiliations:** Institute of Hematology, Union Hospital, Tongji Medical College, Huazhong University of Science and Technology, Wuhan, China

**Keywords:** TEMPI syndrome, monoclonal gammopathy of clinical significance, clinical manifestations, plasma cell-directed treatment, pathogenesis

## Abstract

TEMPI (telangiectasias, elevated erythropoietin level and erythrocytosis, monoclonal gammopathy, perinephric fluid collections, and intrapulmonary shunting) syndrome is a rare and newly defined multisystemic disease, which belongs to “monoclonal gammopathy of clinical significances”. Due to its rarity, the etiology, pathogenesis, and clinical features of this disease remain largely unknown. Owing to its hidden and diverse clinical manifestations, missed diagnosis and misdiagnosis are common. In recent years, as more patients (including three fatal cases) were identified, some special clinical manifestations other than the typical pentad of TEMPI syndrome have been reported. Meanwhile, several studies attempting to identify the pathogenesis of TEMPI syndrome were conducted. In this review, we summarize the reported clinical characteristics of TEMPI syndrome and discuss the current and potential treatment options for patients with TEMPI syndrome, including those with relapsed/refractory disease. Furthermore, we provide an overview of current knowledge on the pathophysiology of TEMPI syndrome.

## Introduction

TEMPI syndrome is an ultra-rare monoclonal gammopathy of clinical significance (MGCS). Coined by Sykes et al. in 2011, the acronym was to describe the shared five clinical features (telangiectasias, elevated erythropoietin level and erythrocytosis, monoclonal gammopathy, perinephric fluid collections, and intrapulmonary shunting) presented in six patients (1). As additional patients were identified and significant clinical improvements were achieved by plasma cell-directed treatment, the disease was classified as a plasma cell neoplasm with associated paraneoplastic syndrome in the World Health Organization Classification of Tumors of Hematopoietic and Lymphoid Tissues, 4th edition, in 2016 (2).

To date, a total of 29 patients with TEMPI syndrome have been identified, including 14 males, 14 females and one not specified. The clinical information of these cases has been retrieved and summarized in **Table 1**. TEMPI syndrome generally has an age of onset in the fifth decade of life without discernable ethnic or geographic predisposition (2). Identification of this condition can be difficult due to its rarity and the constellation of nonspecific presenting signs and symptoms. However, a detailed history and thorough systemic examination might aid diagnosis. Based on the diagnostic criteria proposed by Sykes and colleagues, diagnosis of TEMPI syndrome depended highly on clinical manifestations with telangiectasias, elevated erythropoietin and erythrocytosis, and monoclonal gammopathy as the major criteria. In addition, perinephric fluid collections and intrapulmonary shunting were the minor criteria, and venous thrombosis that was not included in the acronym was listed as other criteria (2). Current treatment strategies focused on eradication of the underlying aberrant plasma cell clone(s) and relieving the TEMPI related clinical features (2, 25). With appropriate treatment, the prognosis of patients was generally good. However, TEMPI syndrome could be fatal, especially for those with aggressive disease or relapsed/refractory disease (17–19). This review summarizes the reported clinical characteristics of TEMPI syndrome, as well as discusses the therapeutic options for patients with TEMPI syndrome, including relapsed/refractory cases. Furthermore, we also provide an up-to-date overview of current knowledge on the pathophysiology of TEMPI syndrome.

**Table 1:** Characteristics of patients reported with the TEMPI syndrome.

No.	Author	Gender	Age	T	Etythrocytosis	EPO	M (g/dL)	P	I	BM-PCs	Others
I	Sykes et al. (1)	Male	42	Yes	Yes	>5000	lgGк (0.7)	Yes	Yes	<10%	Venous thrombosis; Ascites; Plew-effusion; Hepatic hemangioma
2		Female	36	Yes	Yes	>5000	lgGк (0.7)	Yes	Yes	<10%	Venous thrombosis; Spontaneous intracranial hemorrhage
3		Female	39	Yes	Yes	>5000	lgGк (0.7)	Yes	Yes	<10%	Venous thrombosis; Spontaneous intracranial hemorrhage
4		Male	35	NR	Yes	>500	NR	Yes	NR	NR	Hypertension; latrogenic iron-deficient anemia
*5*		Male	*56*	Yes	Yes	Increased	IgG(I)	NR	Yes	<10%	Iatrogenic iron-deficient anemia; Hemanigioma
6		Male	36	NR	Yes	38-86	NR	Yes	NR	NR	Ascites
7	Schroyens et al. (3)	Female	49	Yes	Yes	>8000	lgGк	NR	Yes	NR	NR
8	Mohammadi et al. (4); Jasim et al. (5)	Female	58	Yes	Yes	134	IgAλ (1.4)	Yes	No	10%	Hepatic hemangioma; Hypothyroidism; Hypertension; Diarrhea
9	Kwok et al. (6)	Female	*56*	Yes	Yes	100	IgGλ (3.6)	Yes	NR	10%-15%	Diffuse pains
10	Viglietti et al. (7)	Male	49	Yes	Yes	78	IgAλ (0.2)	Yes	NR	NR	Ascites: Pleural effusion
II	Ryden et al. (8)	Male	50	Yes	Yes	433	lgGк	Yes	Yes	<10%	Glomerulosclerosis
12	Rosado et at al. (9)	Male	49	NR	Yes	5236	lgGк (0.89)	Yes	Yes	7%-18%	Iatrogenic iron-deficient anemia; Venous thrombosis
13	Kenderiam et al. (10)	Female	49	Yes	Yes	8144	IgGк (1.8)	Yes	Yes	5%-10%	Iatrogenic iron-deficient anemia
14	Belizaire et al. (11)	Female	54	Yes	Yes	5000	lgGк (0.8)	Yes	Yes	NR	Iatrogenic iron-deficient anemia Ascites; Bilateral pleural effusions
15	Pascart et al. (12)	Female	*65*	Yes	Yes	>5000	lgGк (2.3)	No	Yes	10.50%	Rheumatoid arthritis
16	Debbaneh et al. (13)	NR	67	Yes	Yes	NR	NR	Yes	Yes	NR	Atrophie blanche
17	Liang et al. (14)	Male	46	NR	Yes	142	igAк (1.23)	Yes	NR	40.60%	NR
18	Shizuku et al. (15)	Male	47	Yes	Yes	4468.3	lgGк	Yes	NR	3.10%	Ascites
19	Manek et a1. (16)	Female	*65*	Yes	Yes	NR	NR	Yes	Yes	NR	Tachycardia
20	Diral et a1. (17)	Female	*56*	Yes	Yes	3656-5728	lgGк (0.98)	Yes	Yes	20%	Right pleural effusion; Retroperitoneal fibrosis; Deep telangiectasia
21	Lor et al. (18)	Male	50	Yes	Yes	NR	lgGк (0.91)	Yes	Yes	NR	NR
22	Guo et al. (19)	Female	70	Yes	Yes	302	lgGк	Yes	Yes	2%	Hypertension; Pericardial effusion
23	Ruan et al. (20)	Female	75	Yes	Yes	>750	lgAλ	Yes	Yes	<10%	NR
24	Liu et al. (21)	Male	47	Yes	Yes	279.76	lgGк (3.175)	Yes	NR	16%	Hypertension
25	Sun et al. (22)	Female	52	Yes	Yes	106	lgGк (0.71)	Yes	Yes	7%	Left pleural effusion; Abdominal effusion; Cyanosis
26		Male	60	Yes	Yes	676.16	igAк (0.51)	Yes	Yes	3%	Pleural effusion: Ascites; Pelvic effusion
27		Male	57	Yes	Yes	741	lgGк (0.96)	Yes	Yes	2.50%	Right pleural effusion; Ascites; Pelvic effusion: Retroperitoneal effusion
28	Strobl et al. (23)	Male	66	Yes	Yes	537	IgGк (0.983)	NR	NR	10%	Cutaneous ulcers: Polyneuropathy; Venous thrombosis: Nail clubbing
29	Kubik et al. (24)	Female	63	Yes	Yes	2265	lgMλ (1.28)	Yes	Yes	15%	NR

T, telangiectasias; EPO, erythropoietin; M, monoclonal gammopathy; P, perinephric fluid collection; I, intrapulmonary shunting; BM-PCs; bone marrow plasma cells; NR; not reported.

## Clinical Features

The typical manifestations of TEMPI syndrome include the pentad of telangiectasias, elevated erythropoietin and erythrocytosis, monoclonal gammopathy, perinephric fluid collections, and intrapulmonary shunting. However, not all features within the acronym are exhibited at the onset of the disease. It seems that erythrocytosis, telangiectasia and monoclonal gammopathy are the initial manifestations, while perinephric fluid collections and intrapulmonary shunting may appear later in the course of disease. Some patients even had no sign of intrapulmonary shunting at first diagnosis (6, 7, 14). There were also other important features not included in the TEMPI acronym, such as venous thrombosis, spontaneous intracranial hemorrhage, and ascites (1, 25).

### Telangiectasias

Telangiectasias are essential components of TEMPI syndrome. Patients typically presented with telangiectasias on the face, upper back and chest, and upper extremities (2). Hands, lips, lower extremities, oral and nasal mucosa, bilateral nares, posterior oropharynx, lunulas of nails were also affected in some patients (15, 16, 20). The classic morphology of telangiectasias was spider angioma or small punctate red stains (18). In those patients who have been tested, dermoscopy showed dilated vessels and red lacuna, while histopathological examination showed no specific changes (20). Although telangiectasias appeared to be confined to the skin, deep telangiectasias distributed in the ascending colon, cranial bone, and dorsal vertebral bodies have been reported recently in one patient (17). Of note, patients with severe skin and deep telangiectasias may concurrently have organ telangiectasias, which may lead to a life-threatening hemorrhage event (17).

### Elevated Erythropoietin and Erythrocytosis

Erythrocytosis is a common laboratory finding of TEMPI syndrome. For this reason, many patients have been erroneously diagnosed with polycythemia vera and initiated on treatment of therapeutic phlebotomy or hydroxyurea (2). In those patients who have been tested, hemoglobin electrophoresis and hemoglobin oxygen affinity testing were normal (2, 17). No mutation of JAK2 gene, beta globin gene, and von Hippel-Lindau gene has been identified (9, 17). In addition, bone marrow morphological findings showed erythroid hyperplasia, without morphologic features associated with myeloproliferative neoplasms (e.g., atypical megakaryocytic hyperplasia, platelet abnormalities, hypercellularity) (9).

Elevated serum erythropoietin was seen in all patients at the time when TEMPI syndrome was diagnosed. The erythropoietin level varied significantly among different patients (ranged from 78 to 8144 mIU/mL) and tended to progressively increase. Significant decrease in erythropoietin level following effective treatment suggests a role of erythropoietin in monitoring therapeutic response (9). In those patients who had stable disease after initial therapy, the increase of the serum erythropoietin appeared to be the most sensitive marker for disease progression and relapse. There are two points that relate to erythropoietin: (a) It remains a question whether the elevation of erythropoietin appears before erythrocytosis or not, because some patients presented with erythrocytosis in the absence of elevated serum erythropoietin at the first clinic visit (17, 26); (b) A potential connection might exist between elevated erythropoietin and telangiectasias or intrapulmonary shunting. Erythropoietin has the effect of promoting angiogenesis, which may aggravate the condition of telangiectasias or intrapulmonary shunting, whereas hypoxia caused by intrapulmonary shunting may in return lead to a further elevation of erythropoietin (27, 28).

### Monoclonal Gammopathy

Monoclonal gammopathy is the hallmark feature of TEMPI syndrome. Among the many reported monoclonal proteins, IgG κ is the predominant type (17 out of 25). IgG λ, IgA λ, IgA κ, IgM λ, and IgE λ were identified in individual cases (**Table 1**) (14, 25). A preponderance of the κ light chain subtype was seen in TEMPI syndrome, with a κ-to-λ ratio of 19: 5. Serum level of monoclonal protein in reported cases was typically <3.0 g/dL (ranged from 0.0815 to 3.6 g/dL). The free light chains, free light chain ratios, and Bence-Jones proteinuria testing were generally normal. Exceptions were found in individual cases. The monoclonal protein reached 3.6 g/dL in patient 9 (**Table 1**) and the serum free light chain ratios skewed in 4 cases.

Patients with TEMPI syndrome typically had <10% plasma cells in the bone marrow and lacked myeloma-defining events. Seven patients were reported to have > 10% bone marrow plasma cells (**Table 1**). Especially, one patient with relapsed MM showed unexplained secondary erythrocytosis, elevated erythropoietin, and severe bilateral perinephric fluid collections and was diagnosed with TEMPI syndrome. His bone marrow biopsy showed clonal plasma cells up to 40.6% (14). Interestingly, after review of the literature, we have identified four patients of MM or smoldering multiple myeloma (SMM) with unexplained secondary erythrocytosis. Two patients were diagnosed erythrocytosis years before the detection of MM, and the others were diagnosed simultaneously with secondary erythrocytosis and MM/SMM (29–32). Their monoclonal proteins have been reported to be IgG κ or IgA λ, which were both seen in TEMPI syndrome. However, no telangiectasias, perinephric fluid collections, or intrapulmonary shunting was mentioned in these patients. Taken together, there remain two questions: (a) Whether the plasma cell percentages in some patients with TEMPI may reach the level of SMM or MM as the disease progresses; (b) Whether there are more MM patients complicated with undiagnosed TEMPI Syndrome.

### Perinephric Fluid Collections

Perinephric fluid collections appeared bilaterally or unilaterally in patients with TEMPI syndrome. The fluid would increase or decrease as the patient’s condition changed. When reaching a certain level, perinephric fluid collections might lead to abdominal fullness, flank pain, nausea, hypertension, or abdominal mass. Perinephric fluid collections could be detected with ultrasound, computed tomography (CT) or magnetic resonance imaging (MRI). When examined, the fluid was sterile and had the same chemical composition as serum with no/little cells and small amounts of protein, cholesterol, triglycerides, and chylomicron (1, 4).

### Intrapulmonary Shunting

Intrapulmonary shunting is another typical manifestation of TEMPI syndrome. The progressively increasing shunting index could result in a manifestation of hypoxemia. Cyanosis and dyspnea on exertion were both common reasons for TEMPI patients to require the initiation of treatment. Some patients even needed a continuous supplement of high-concentration oxygen (10). ^99m^Tc macro-aggregated albumin scintigraphy was best for the quantitative detection of intrapulmonary shunting (2).

### Others

Except for telangiectasias, other skin changes included atrophie blanche, stasis dermatitis, hemangioma-like lesions, red macules, and purple angiomatous papule (13, 17, 18). Nail change included clubbing (23). Cyanosis due to intrapulmonary shunt could also occur (22). Cutaneous ulcers located at bilateral lower legs were found in three patients (13, 23). The rapid healing of ulcerations after treatment of bortezomib suggested a potential role of monoclonal gammopathy in the pathogenesis of cutaneous ulcers (23). The incidence of serous cavity effusions other than perinephric fluid collections was relatively common in TEMPI patients (22). Of the 29 patients, ten suffered from serous effusion (7, 11, 15, 22, 26). It often affected more than one cavity (6/10). The two most common sites were abdominal (8/10) and pleural (7/10). It is interesting that a mild sensation of abdominal fullness caused by ascites was the main reason for one patient to seek medical help (15). The etiology of the serous effusion remains unclear. However, the concurrent increase in left pleural effusion and left perinephric fluid collection (26), a sterile transudative fluid of pleural effusion, and the improvement of pericardial effusion after plasma cell-directed therapy suggest that serous effusions and perinephric fluid collections may share a pathophysiologic association. The risk of vascular diseases may increase in TEMPI syndrome (26). Five patients with TEMPI syndrome developed venous thrombosis, two experienced the complication of spontaneous intracranial hemorrhage without identifiable arteriovenous malformations, and two suffered from liver hemangiomas (1, 4, 5). Additionally, gastrointestinal arteriovenous malformations were detected in one patient (2). Other diseases including rheumatoid arthritis, hypertension, pulmonary hypertension, axonal polyneuropathy, heart disease, hypothyroidism, and focal segmental glomerulosclerosis were also identified in individual cases (5, 8, 12, 23).

Similar with POEMS syndrome, diagnosis of TEMPI syndrome depends highly on clinical manifestations. In 2020, Sykes et al. proposed diagnostic criteria for TEMPI syndrome. Telangiectasias, elevated erythropoietin and erythrocytosis, and monoclonal gammopathy were listed as the major criteria. Perinephric fluid collections and intrapulmonary shunting were listed as the minor criteria (2). However, no concrete criteria for the definition of the disease have been made. Due to the constellation of nonspecific symptoms, missed diagnosis and misdiagnosis were commonly made in TEMPI syndrome. Of the reported cases, the average time from symptoms to diagnosis was 10 years (ranging from 2 months to 41 years). However, with a detailed history, physical exam, and thorough workup that includes monoclonal protein examinations (protein electrophoresis, immunofixation, and serum free light chains), EPO levels, a bone marrow biopsy, ^99m^Tc-labeled macroaggregated albumin lung perfusion scintigraphy, and computed tomography scans of abdomen, this syndrome can be differentiated from other conditions.

## Management

Because of the rarity of TEMPI syndrome, no specific treatment guideline has been developed. Evidence for treatment in TEMPI syndrome was largely limited to case reports. In general, therapy for TEMPI syndrome was characteristically divided into two components: firstly, eradication of the underlying aberrant plasma cell clone(s) through plasma cell-directed treatment as used in treating multiple myeloma, and secondly, symptomatic treatment for relieving TEMPI related clinical features (erythrocytosis, perirenal effusion, hypoxia, etc.).

### Plasma Cell-Directed Therapy

As a MGCS, TEMPI syndrome responds to plasma cell-directed therapy rapidly and remarkably. Bortezomib, daratumumab, lenalidomide, high dose melphalan and autologous stem cell transplantation (ASCT) have been proven effective, leading to at least partial responses with good outcomes for most TEMPI patients (3, 10, 14, 33).

#### Proteasome Inhibitors

Bortezomib is a reversible first-generation proteasome inhibitor (PI) commonly used in TEMPI syndrome, which is effective and well-tolerated (3). It may not only promote the death of abnormal plasma cells, but also directly resolve part of the clinical features by affecting the function of HIF-1α (34). For most patients who received treatment of bortezomib-based regimen, clinical improvement was achieved. Of 13 patients with available hematological response, 3 showed complete response, 7 had partial response, and 3 showed primary refractory. Caution should be exercised with bortezomib, owing to the risk of peripheral neuropathy. When administered with bortezomib, two patients developed neuropathy, one developed painful grade 1 peripheral neuropathy, and one presented with gastric retention for the gastrointestinal neurotoxicity who eventually died from multiple organ failure caused by infection (5).

#### Immunomodulatory Drugs

As the first generation of immunomodulatory drugs (IMiDs), thalidomide has been proven ineffective for TEMPI patients (1). A total of three patients receiving the treatment of thalidomide have progressed. Unlike thalidomide, the second generation of IMiDs, lenalidomide, has been reported for the treatment of 4 patients and shown efficacy. All of them were refractory to bortezomib or relapsed after bortezomib treatment. After the usage of lenalidomide, 2 showed at least partial response, one patient showed good clinical improvement though his erythropoietin increased, and one patient had disease progression (11, 14, 18, 33). However, it was not clear whether the good clinical response to lenalidomide resulted from the direct control of aberrant plasma cell clone(s) or the immunomodulatory effect.

#### Autologous Stem Cell Transplant

ASCT was used as the salvage treatment for three patients who had inadequate responses or whose diseases progressed after treatment of PI or IMiDs. Among the three patients, one achieved complete response and one managed stable disease (10, 33). However, the other one developed unexpected complications of grade IV mucositis and severe mucosal bleeding, and unfortunately died from a progressive respiratory failure (17). Therefore, though ASCT may be effective for some patients, significant risks remain. During the management of TEMPI patients, it is necessary to compare relative risks and benefits of ASCT to those of other treatments.

#### Anti-CD38 Monoclonal Antibody

Daratumumab is a human anti-CD38 monoclonal antibody with good clinical efficacy and safety for relapsed/refractory multiple myeloma. Daratumumab monotherapy was reported to be effective in two patients with diseases that relapsed or was refractory to bortezomib, lenalidomide, and ASCT (33). For the reason of its efficacy and tolerance, daratumumab has been recommended as initial treatment for TEMPI syndrome (2). Nevertheless, a recently-reported patient was refractory to daratumumab and died of disease progression (18).

### Other Potential Treatments for Relapsed/Refractory TEMPI Syndrome

Although most patients with TEMPI syndrome responded well to bortezomib, daratumumab, lenalidomide, high dose melphalan and autologous stem cell transplantation, two deaths due to resistance to current treatment strategies were reported (17, 18). It highlighted the need for additional therapies. Recently, effective therapeutic strategies for relapsed/refractory MM (RRMM) have been developed, including anti-BCMA CAR-T (chimeric antigen receptor T-cell) therapy, and new generation of PIs and IMiDs (35–38). As the eradication of underlying aberrant plasma cell clones and M protein is essential in the treatment of TEMPI syndrome, the successful therapeutic strategies for RRMM could be borrowed for the management of relapsed/refractory TEMPI syndrome. However, further study should be conducted to verify the safety and feasibility of those treatments.

### Supportive Care

The management of individual symptoms is of importance in clinical practice. Before diagnosed, therapy for TEMPI patients was almost invariably to relieve TEMPI related symptoms, such as erythrocytosis, perirenal effusion, and hypoxia. Supportive care is invaluable, because the plasma cell-directed therapeutic modalities such as chemotherapy, autologous transplantation, and anti-CD38 monoclonal antibody cannot lead to an immediate benefit to patients and may also cause drug-related complications. Hypoxia caused by intrapulmonary shunting requires oxygen inhalation, even continuous supplementary oxygen (6). Perinephric fluid aspiration or drainage has been effective for patients with severe or increasing perinephric fluid collections (7). In the case of other clinical symptoms and treatment-related adverse effects, regular monitoring and appropriate intervention are necessary.

## Follow−Up and Outcomes

According to the reported cases, TEMPI syndrome was slowly and steadily progressive. The median time from disease onset to diagnosis was about 10 years. Treatment responses to plasma cell-directed therapy have been rapid and remarkable, with clinical improvement rate > 80% after first-line treatment (bortezomib-based regimen). Even for patients who relapsed or were refractory to bortezomib-based regimen, the prognosis was sometimes good: most of the patients responded to ASCT, lenalidomide, or daratumumab (10, 14, 33). However, due to the rarity and the merely 10-year history of the disease, there was little information about the outcomes of TEMPI syndrome provided by large series and long-term follow-up studies. A 54-year-old female achieved a complete remission after eight cycles of intravenous bortezomib and had maintained progression free survival for more than 6 years. Subsequently, she still achieved a complete response following treatment with daratumumab when disease relapsed (33).

In the case of three patients reported by our group (**Table 1**) (22), patient 25, a 56-year-old woman, received six cycles of bortezomib/cyclophosphamide/dexamethasone (VCD) and achieved a very good partial response. She has remained progression-free for over 3 years since her last cycle of VCD. Patient 26, a 60-year-old man, received two cycles of bortezomib/dexamethasone (VD). After treatment, he had a partial response, but developed painful grade 1 peripheral neuropathy. He was then treated with regimen of ixazomib/dexamethasone (IXD) for one cycle, which was discontinued because of the intolerance of the patient to ixazomib. Thereafter, the patient received twelve cycles of lenalidomide/dexamethasone (Rd) and had a complete remission after two cycles of treatment. Till now, he has remained in complete remission for about 2 years. Patient 27, a 57-year-old man, received four cycles of Rd followed by six cycles of VCD. He had remained a stable disease until disease progression at the end of the last cycle of VCD. He was then started on the regimen of melphalan/dexamethasone/thalidomide (MDT) and achieved a stable disease again. He has received seven cycles of MDT, and the treatment is ongoing.

Although the prognosis of TEMPI syndrome was generally good with appropriate treatment, it could be fatal, especially for those with aggressive disease or relapsed/refractory disease. To date, three deaths due to TEMPI syndrome have been reported (17, 18). Of note, all three patients had the same type of monoclonal protein as IgG Kappa and were primary refractory to or intolerant of bortezomib. Meanwhile, telangiectasias were persistent in all three cases, though some clinical features improved after treatment. These common characteristics suggested that patients who are primary refractory to or intolerant of bortezomib might have poor outcomes. Moreover, the persistence of skin telangiectasias after treatment could be considered as an index of aggressive disease, and therefore, as a prognostic factor for poor outcomes for TEMPI patients (17). Additionally, given that one fatal case was 70-year-old and age is an independent poor prognostic feature for MM and POEMS, we suspected that elderly or frail patients with TEMPI syndrome might also have poor outcomes.

## Pathogenesis

The pathogenesis of TEMPI syndrome remains poorly understood. The complete relief of the symptoms with effective plasma cell–directed therapy has implicated a central role of either the abnormal plasma cell clone or monoclonal gammopathy in the pathogenesis of TEMPI syndrome (2). A few attractive hypotheses on the pathogenesis have been postulated.

Firstly, a single or more circulating factors (chemokine or cytokine) secreted by monoclonal plasma cells may activate or inactivate a complex cytokine process in the causation of the varied symptom constellation. Macrophage migration inhibitory factor (MIF) is of interest for it can explain most of the constellation of symptoms in TEMPI syndrome and has been identified elevated in 3 patients. In addition, the level of serum MIF significantly decreased following effective treatment with the bortezomib-based regimen (22). The disturbance of TGF-β signaling pathway may also be an intriguing explanation for its pivotal role in neoangiogenesis, vessel maturation and stabilization (39). Previous studies have proven that the TGF-β signaling pathway could lead to the appearance of telangiectasias and arteriovenous malformations, and contribute to the formation of serous effusion (39, 40). Judging from the relevance between serous effusions and perinephric fluid collections, TGF-β signaling pathway might also be involved in the formation of perinephric fluid collections.

Secondly, an autoimmune disorder involved in the pathogenesis of TEMPI syndrome may be mediated by the interaction between monoclonal antibody and a nonprotein or an intracellular antigenic target (2). To verify this hypothesis, techniques including immunofluorescence of fresh-frozen normal tissue samples, immunohistochemistry of fixed normal tissue samples, immunoblotting of denatured tissue lysates, and peptide and protein microarrays have been applied by Sykes et al. However, no convincing antigenic target has been identified (2). It is also intriguing to hypothesize that the deposition of monoclonal immunoglobulin or its fragments may perturb the oxygen-sensing mechanism and in some way enhance the function of HIF-1α, ultimately driving the expression of erythropoietin (2, 25).

Recently, a few attempts have been made to identify possible genetic abnormalities associated with this disease. Rosado et al. performed gene expression analysis (RNA sequencing) and mutational analysis (whole-exome sequencing) of bulk-sorted plasma cell populations in three patients (41). Diral et al. performed exome sequencing analysis of a paired blood/germline (whole blood samples and buccal swabs) from one patient (17). However, no molecular genetic abnormality associated with TEMPI has been identified from these studies. In a recent study by our group, we used whole-genome sequencing (WGS) and identified some somatic mutations of interest in monoclonal PCs in one patient with TEMPI. The genetic variants include complex structural variants of chromosome 2 within regions where some putative oncogenes reside, such as MYCN (2p24.3), REL (2p16.1), BCL11A (2p16.1), BCL2L11 (2q13), MAPK4 (2p22.1), and TP53I3 (2p23.3), and duplication of 22q11.23 where the gene of MIF is located (22). However, further studies are needed to verify whether these findings were recurrent or incidental events.

## Conclusion

In summary, TEMPI syndrome is an ultra-rare, but treatable MGCS. Making the diagnosis can be a challenge. A detailed history (longstanding and unexplained erythrocytosis), physical examination (skin changes), and appropriate testing-analysis is helpful. Current treatment approaches make a good prognosis possible. Once the diagnosis is made, application of supportive care and anti-plasma cell treatments is essential. The pathogenesis of TEMPI syndrome remains poorly understood. Further researches are required to establish more concrete criteria for diagnosis and treatment, identify the prognostic features of TEMPI syndrome, and illustrate the exact underlying pathophysiology. Given the rarity of TEMPI syndrome, global cooperation is essential.

## Author contributions

CS, YH, and JX contributed to the paper’s conception and design; JX, WL, BZ, and FZ contributed to the literature search; JX, WL, and FF wrote the original draft, CS and YH reviewed and revised the paper. All authors contributed to the article and approved the submitted version.

## Funding

This work was supported by the National Natural Science Foundation of China, 81974007 (C.S.) and 82000223 (J.X.).

## Conflict of Interest

The authors declare that the research was conducted in the absence of any commercial or financial relationships that could be construed as a potential conflict of interest.

## Publisher’s Note

All claims expressed in this article are solely those of the authors and do not necessarily represent those of their affiliated organizations, or those of the publisher, the editors and the reviewers. Any product that may be evaluated in this article, or claim that may be made by its manufacturer, is not guaranteed or endorsed by the publisher.
